# Distinct Phyllosphere Bacterial Communities on *Arabidopsis* Wax Mutant Leaves

**DOI:** 10.1371/journal.pone.0078613

**Published:** 2013-11-05

**Authors:** Eva E. Reisberg, Ulrich Hildebrandt, Markus Riederer, Ute Hentschel

**Affiliations:** University of Würzburg, Department of Botany II, Julius-von-Sachs-Institute for Biological Sciences, Würzburg, Germany; Argonne National Laboratory, United States of America

## Abstract

The phyllosphere of plants is inhabited by diverse microorganisms, however, the factors shaping their community composition are not fully elucidated. The plant cuticle represents the initial contact surface between microorganisms and the plant. We thus aimed to investigate whether mutations in the cuticular wax biosynthesis would affect the diversity of the phyllosphere microbiota. A set of four *Arabidopsis thaliana eceriferum* mutants (*cer*1, *cer*6, *cer*9, *cer*16) and their respective wild type (Landsberg *erecta*) were subjected to an outdoor growth period and analysed towards this purpose. The chemical distinctness of the mutant wax phenotypes was confirmed by gas chromatographic measurements. Next generation amplicon pyrosequencing of the bacterial communities showed distinct community patterns. This observation was supported by denaturing gradient gel electrophoresis experiments. Microbial community analyses revealed bacterial phylotypes that were ubiquitously present on all plant lines (termed “core” community) while others were positively or negatively affected by the wax mutant phenotype (termed “plant line-specific“ community). We conclude from this study that plant cuticular wax composition can affect the community composition of phyllosphere bacteria.

## Introduction

Above ground plant surfaces are inhabited by diverse microbial communities, of which few bacterial phyla predominate in the plant phyllosphere [1], [2]. Environmental factors like sunlight irradiation, season, geographic location and sampling site are recognized as important factors in shaping the phyllosphere microbiota [3]–[6]. In addition, the phyllosphere microbial community has been reported to be plant species-specific [6]–[8]. Moreover, leaf properties, such as size, colour, mineral content, the presence of veins, stomata and surface appendages (including trichomes and hydathodes) have been hypothesized to affect microbial community composition [1], [9]. However, the precise mechanisms determining the composition of microbial communities on leaf surfaces remain largely unclear.

The plant cuticle is the first contact point for immigrating microorganisms and serves both as a protective barrier for the plant and as a habitat for microorganisms. The outermost layer of the cuticle is composed of long-chain fatty acids and derivatives thereof, which are termed cuticular waxes. Pentacyclic triterpenoids and sterols are additional components of the waxes in many plant species. The cuticular wax composition is plant species-specific and can differ between plant organs and developmental stages [10]–[12]. Waxes contribute to the transpiration barrier properties of the plant cuticle [13]–[15] and have UV absorbing as well as self-cleaning effects [16], [17]. Specific effects of plant cuticular wax constituents on plant leaf colonisation of pathogenic fungi were described [18]–[20]. Only few studies have addressed the effects of cuticular waxes on phyllosphere bacteria. It was shown by spray inoculation of maize *glossy* mutants that leaf surface waxes can affect bacterial leaf colonisation [21]. In a different study, *cer*1 overexpressing *A. thaliana* plants displayed an increased susceptibility to bacterial and fungal pathogens [22]. To our knowledge, the influence of leaf surface waxes on bacterial colonisation under natural conditions has not been investigated.

In the present study we investigated a set of four *A. thaliana eceriferum* mutants with pronounced differences in the epicuticular leaf wax composition as well as the respective wild type [23]. These plants are known to contain mutations in genes encoding a putative very-long-chain-aldehyde decarbonylase (*CER1*), a β-ketoacyl-CoA-Synthase (*CER6*), as well as a putative E3 ubiquitin ligase (CER9), while the mutated gene in *cer16* remains to be identified. The corresponding wild type genes contribute through different mechanisms to the correct formation of the very long chain lipid components of the cuticle layer during wax biosynthesis [11].

The aim of the present study was to investigate whether plant wax mutant phenotypes would affect the bacterial community composition of naturally colonizing phyllosphere bacteria. The leaf associated bacterial communities of the five plant lines were examined by denaturing gradient gel electrophoresis (DGGE) and amplicon pyrosequencing. We present findings that suggest that plant leaf cuticular waxes are one important factor shaping the community composition of phyllosphere bacteria.

## Materials and Methods

### Plants and cultivation conditions

The A. *thaliana* wild type Landsberg *erecta* (Ler) and four *eceriferum* (*cer*) mutants [24] in Ler background (*cer*1, *cer*6, *cer*9, *cer*16) were obtained as seeds from the European Arabidopsis Stock Centre (NASC, http://arabidopsis.info/). Seeds were surface-sterilized as described in Reisberg et al. [25] and plants were grown in 7.5 cm pots on initially autoclaved soil (Einheitserde P (Patzer, Sinntal-Jossa, Germany), Vermiculite, Sand (ratio 10∶0.5∶0.5)). The soil was autoclaved in order to prevent colonization with bacteria from pot soil rather than from the environment. The plants were grown for 8–9 weeks under controlled climate conditions in a growth cabinet in order to ensure simultaneous, sufficient germination of seeds and production of leaf biomass. The conditions were set at a 9-h day period at 21°C and a 15-h night period at 18°C, a photon flux density of 70 *µ*mol m^–2^ s^–1^, and a relative humidity of 70%. After this period the plants were transferred outdoors besides the greenhouses of the Botanical Garden Würzburg, Germany. The plants remained exposed to the environment for 4 weeks during April-June of the years 2010, 2011 (microbial community analyses) and 2012 (wax analyses) and were watered with rain water as necessary. All analyses were conducted with at least triplicate samples for each plant line.

### Wax analysis

Epicuticular waxes were extracted by immersion of 10 to 20 rosette leaves which were pooled from two to three plants in 10 ml of chloroform for 30 sec at room temperature (3 such pools were generated per plant line). 5 µg n-tetracosane (Fluka, Sigma-Aldrich, Steinheim, Germany) were added to the extraction solution as an internal standard. The solvent was evaporated under nitrogen flow and the remaining lipids were treated with Bis-N,O-(trimethylsilyl)trifluoroacetamide (Macherey-Nagel, Düren, Germany) at 70°C for 30 min to transform hydroxyl groups into trimethylsilyl groups. Subsequently, the samples were re-dissolved in 100 µl of chloroform and analysed by gas chromatography coupled with flame ionization detection (GC/FID; 5890 Hewlett Packard Series II, Agilent Technologies, Santa Clara, CA, USA) for quantification, and mass spectrometry (GC/MS; 6890N & MSD 5973; *m*/*z* 50–750, Agilent Technologies) for compound identification. After on-column injection a temperature protocol of 2 min at 50°C, 40°C min^−1^ to 200°C, 2 min at 200°C, 3°C min^−1^ to 320°C, and 30 min at 320°C was carried out. For GC/FID a ZB-1 column with 30 m length, 0.32 mm inner diameter and a film thickness of 0.1 µm (Zebron™, Phenomenex, Aschaffenburg, Germany) was applied. Hydrogen was used as carrier gas with the following pressure protocol: 5 min at 50 kPa, 3 kPa m^−1^ to 150 kPa, and 50 min at 150 kPa. For GC/MS, a DB-1HT column of 30 m length, 0.32 mm inner diameter, and a film thickness of 0.1 µm (Agilent Technologies) was used. Helium was used as carrier gas using the same pressure protocol except that the final 150 kPa step was for 40 min. Analysis of the spectra was carried out using the HP ChemStation software (Agilent Technologies). The total leaf area was determined by counting pixels (Adobe Photoshop Systems, Dublin, Ireland) of scanned images of the extracted leaves in comparison to a defined reference area and multiplying it by 2 considering abaxial and adaxial leaf surfaces.

### Phyllosphere bacterial community sampling

5–10 leaves per plant were removed with sterile forceps and immersed in 10 ml of potassium phosphate buffer (pH 7). Bacterial communities were detached by a 7 min sonification step in an ultrasonic cleaning bath (Sonorex Super RK 514 BH, Bandelin Electronics, Berlin, Germany), [26]. After removal of the leaves, the remaining bacterial suspension was centrifuged for 20 min at 16,000 x g (Microcentrifuge 5415R, Eppendorf, Hamburg, Germany) and 4°C. The supernatant was discarded and the bacterial cells were resuspended in the remaining liquid. DNA was extracted using the MasterPure Complete Nucleic Extraction Kit (Epicentre Biotechnologies, Madison, Wisconsin).

### Denaturing Gradient Gel Electrophoresis (DGGE)

PCR was performed using the universal bacterial primers 341f+GC/907r targetting the 16S SSU rRNA gene [27]. The KAPA2G™robust PCR-Kit (Peqlab, Erlangen, Germany) was used with buffer A, 2 µg bovine serum albumin, 0.2 mM dNTPs, 2 µM of each primer and 0.4 U polymerase per reaction. The PCR conditions were as follows: one 95°C step for 2.5 min and 35 cycles of 95°C for 30 sec, 60°C for 30 sec, 72°C for 20 sec. DGGE-PCR products were visually inspected on agarose gels. The remaining volumes (44 µl) of the PCR-products were subsequently loaded on gels containing a denaturing gradient from 0 to 80%. Gels were run in a DCode™ apparatus (Bio-Rad Laboratories, Munich, Germany) at 200 V for 4.5 h. The gels were stained with ethidium bromide and visualized on a Gel Doc XR device (Bio-Rad Laboratories, Munich, Germany). DGGE gel images were analysed using the QuantityOne® 1D-Analysis Software (Bio-Rad Laboratories, Munich, Germany). Gel bands were marked automatically as well as manually with the help of a DGGE marker lane (Wako Chemicals GmbH, Neuss, Germany). Gel-to-gel comparisons were accomplished by use of the marker lane and wild type samples which were included on each gel. We included ten DGGE gels in the comparison, each containing 3 wild type and 3 mutant samples, each in two technical replicates (12 lanes per DGGE run). In total 4 biological replicates of all plant lines were investigated. Each biological replicate was represented by 10–18 technical replicates (for the wild type) or by 1–5 technical replicates (for mutant samples). The information on the presence or absence of bands from the different gels was combined into a binary matrix, which was then subjected to a nonmetric multidimensional scaling analysis (NMDS) as well as an analysis of similarity (ANOSIM) with the Jaccard similarity index using the PAST statistical analysis software [28].

### Amplicon sequencing

The amplicon libraries were generated on the same DNA samples and using the same primer set (341f/907r, [27]), thus amplifying the hypervariable regions V3, V4 and V5. The primers were supplemented with the 454-sequencing adaptors A and B and an additional multiplex identifier sequence for each sample at the forward primer. For these reactions the KAPAHiFi™ PCR-Kit (Peqlab, Erlangen) was used with buffer A, 0.3 mM dNTPs, 0.3–0.4 µM of each primer and 0.5 U polymerase per reaction. PCR conditions were as follows: one 98°C step for 3 min and 29–38 cycles of 98°C for 20 sec, 56/60°C for 20 sec, 72°C for 20 sec. The amount of template DNA was ∼65–70 ng for each reaction. The amplification was visually checked by agarose gel electrophoresis and the optimal cycling conditions were chosen to result in equally intensive bands for the samples analyzed. This adaption was performed for each of the 15 primers, as their reaction efficiency was slightly variable, likely as a result of the individual multiplex identifier sequences. 5–12 independent PCR reactions were pooled for each amplicon library and purified using the NucleoSpin Extract II Kit (Macherey-Nagel, Düren, Germany). The nucleic acid content was measured with a NanoDrop 1200 photometer (Peqlab, Erlangen, Germany). Purification success and quality of the PCR amplicons were examined using the Experion™ DNA 1K Analysis Kit (Bio-Rad Laboratories, Munich, Germany). One µg of each PCR product was sent to LGC Genomics (Berlin, Germany) for equimolar pooling of the amplicon libraries, emulsion PCR and sequencing on the FLX+ Titanium sequencing platform. 15 barcoded amplicon libraries, representing three biological replicate samples per plant line, were sequenced on a quarter picotiter plate.

### Amplicon data processing and statistical analyses

Sequence analysis was performed using the QIIME pipeline v 1.4 [29]. Raw sequences were deposited in the NCBI Sequence Read Archive (SRP018905). Initially, raw sequences were sorted according to their barcodes and quality-filtered at a mean quality level of 25. Reads shorter than 300 bp or with mismatches in the primer or barcode, as well as sequences containing ambiguous bases or homopolymers longer than 6 bases were excluded. Data were denoised based on the original flowgrams using the denoise_wrapper.py script of QIIME. Operational Taxonomic Units (OTUs) were picked at 97% sequence similarity level using UCLUST [30] and the “—optimal” option. Representative sequences of each OTU were aligned against the Greengenes core dataset [31] with the PyNAST algorithm [32]. Chimeras were detected using ChimeraSlayer [33] with the Greengenes core set [31] as a reference and excluded from downstream analysis. Taxonomy to bacterial family level was assigned using the RDP classifier [34] at a 0.8 confidence threshold. Sequences that were assigned to the *A. thaliana* chloroplast 16S rRNA gene as well as singletons were removed from the dataset. The processed sequence files are available as Supplementary Information (Sequence File S1–5).

For diversity computation, all samples were rarified to the sample with the lowest sampling effort (2340 sequences). Diversity indices, Pilou evenness and richness estimators were calculated using the ‘vegan’ package v 2.0–5 of R [35]. Rarefaction curves were calculated using the alpha_rarefaction.py script of QIIME. Beta diversity calculations were carried out on a binary bacterial “resident community” dataset extracted from a rarified OTU table (see explanation in results for further details). With this dataset an NMDS plot and analysis of similarity (ANOSIM) with the Jaccard similarity index were conducted using the PAST software [28]. UniFrac distance based clustering with Unweighted Pair Group Method with Arithmetic Means (UPGMA) was done by using the jackknived_beta_diversity.py script of QIIME with default settings. A Mantel test comparing the resident communities of all plant lines with the corresponding wax profiles was conducted using the Jaccard similarity index for the OTU matrix and the Euclidean similarity index for the wax profiles with the PAST software.

For the presentation of the plant line resident communities genus level classification was achieved by a repeated alignment and assignment to the SILVA_r108 database (http://www.arb-silva.de/) using the same methods as above. Chimera checking was repeated with the SILVA_r108 core sequences (http://www.arb-silva.de/) as reference. Chimeras that were detected only within one reference dataset (Greengenes or SILVA) were maintained and labelled accordingly or removed. Venn diagrams as well as a heatmap with corresponding dendrogram (complete linkage clustering, distance method “binary”) of the resident community OTUs were generated with the ‘gplots’ package v 2.11.0 of R [36].

## Results

### Cuticular wax analyses

**Figure 1:**
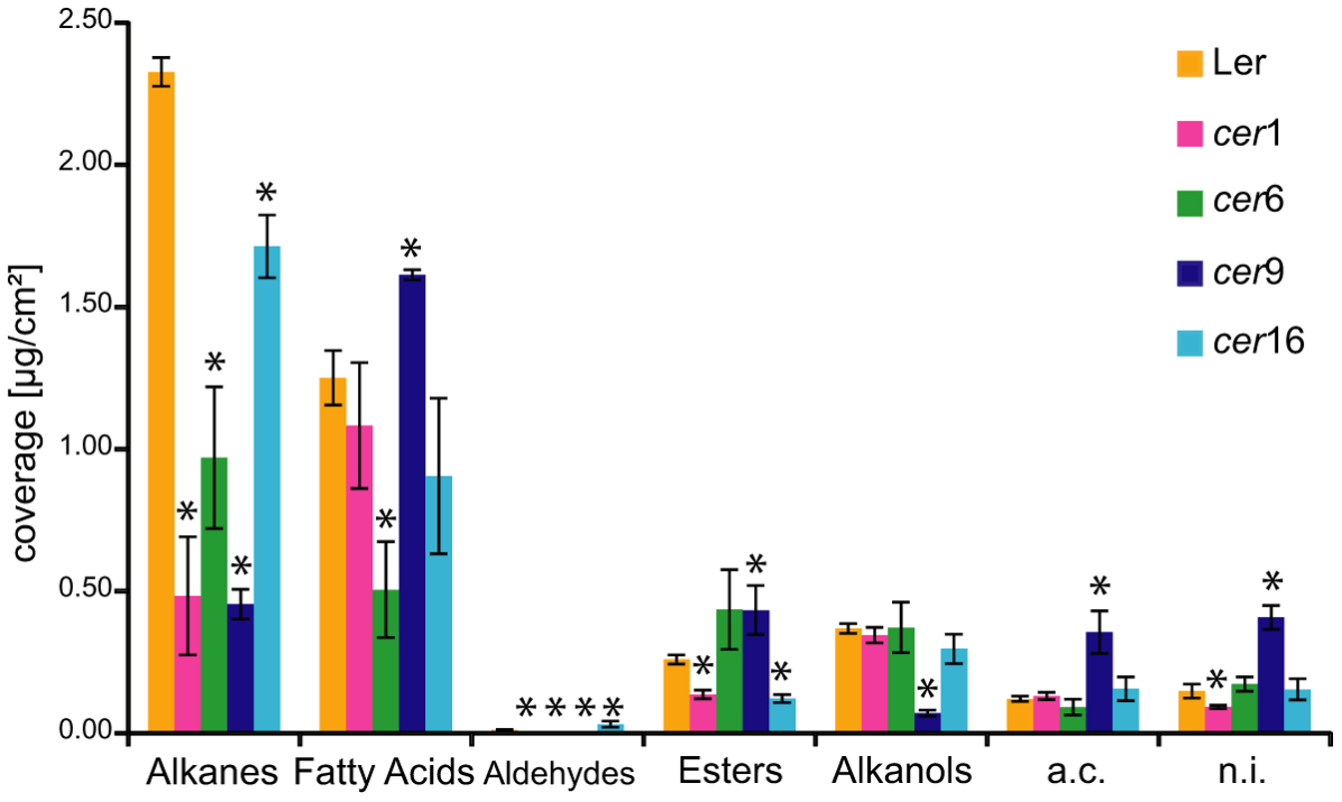
Analyses of *cer* mutant cuticular waxes. Cuticular wax extracts of five 14-week-old *A. thaliana* lines (*cer*1, *cer*6, *cer*9, *cer*16, Ler) grown partially under outdoor growth conditions were analysed. The main compound classes were alkanes, fatty acids, wax esters and alkanols (µg cm^−2^ ± SD; n = 3) Abbreviations: a.c.  =  additional components (combining sterols, triterpenoid-like compounds, C_29_ ketone, secondary alkanols and monoglycerides of fatty acids), n.i.  =  not identified compounds.

The cuticular wax coverage of four *cer* lines in a Ler background of *A. thaliana* was analysed by GC/MS and GC/FID for identification and quantification of wax components. The plants were 13 weeks old and had been exposed to a four weeks outdoor growth period prior to analysis. Mean total wax amount for Ler wild type leaf extracts was 4.49±0.35 µg cm^−2^ leaf area (n = 3). The wax amount in the mutant leaf extracts was overall lower and ranged from 2.28±0.06 µg cm^−2^ (*cer*1), 2.55±0.60 µg cm^−2^ (*cer*6), 3.34±0.19 µg cm^−2^ (*cer*9), to 3.38±0.51 µg cm^−2^ (*cer*16). The composition of the cuticular waxes was different between the five plant lines. The mutant lines exhibited significantly lower total amounts of alkanes in the cuticular leaf wax extracts than the wild type. While Ler leaves contained 2.3±0.05 µg cm^−2^ alkanes, the leaf alkane contents in mutants were 0.48±0.21 µg cm^−2^ (*cer*1), 0.97±0.25 µg cm^−2^ (*cer*6), 0.45±0.05 µg cm^−2^ (*cer*9), and 1.71±0.11 µg cm^−2^ (*cer*16) (Fig.1; Fig.S1). The chain length distribution in the alkane fraction was comparable between all plant lines investigated, with the main components being C_31_, C_29_ and C_33_ alkanes.

The wild type leaf wax extracts contained high amounts of free fatty acids (1.25±0.1 µg cm^−2^), with dominating carbon numbers 32 and 34 (Fig.1; Fig.S1). The mutants *cer*1 and *cer*16 exhibited nearly similar amounts of fatty acids and a similar chain length distribution as the wild type (1.08±0.22 µg cm^−2^ and 0.91±0.27 µg cm^−2^, respectively). Fatty acids in the *cer*6 mutant leaf wax extracts were significantly reduced by half compared to the wild type and showed considerably lower amounts of C_32_ and C_34_ fatty acids. On the other hand, *cer*9 leaf wax extracts contained significantly increased amounts of fatty acids (1.61±0.02 µg cm^−2^). The C_24_ and C_26_ fatty acids were about one order of magnitude higher (0.30±0.02 µg cm^−2^ of C_24_ fatty acid and 0.59±0.01 µg cm^−2^ of C_26_ fatty acid) and the C_28_ fatty acid was about twice the amount of the wild type. However the C_32_ and C_34_ fatty acids amounted to only one third and one fifth of the content in wild type wax extracts, respectively.

Aldehydes were detected in very low amounts (≤0.01 µg cm^−2^) in all plant lines, except *cer*16 where 0.03±0.01 µg cm^−2^ aldehydes were detected (Fig.1). Considerable amounts of alkyl esters were identified in all plant lines, with *cer*6 (0.44±0.14 µg cm^−2^) and *cer*9 mutants (0.43±0.09 µg cm^−2^) showing highest amounts. Primary alkanols were found on wild type and on *cer*1, *cer*6, *cer*16 mutants in moderate amounts (0.30 – 0.37 µg cm^−2^), while the *cer*9 mutant wax extracts displayed only 0.07±0.01 µg cm^−2^. Branched chain isoforms of C_28_, C_30_, C_31_, C_32_ and C_34_ alkanols were detected in all plant lines except *cer*16 (Fig.S1).

Several compounds were summarized as “additional components”. These are sterols, C_29_ ketone, C_29_ secondary alcohol and a compound class resembling the mass spectra of triterpenoids, all of which were detected in similar amounts in all plant lines. One component class of this category was found in high amounts in *cer*9 (Fig.1). A comparison with published mass spectra [37] resulted in a preliminary identification as monoglycerides of fatty acids, mainly of the chain lengths of C_24_, C_26_ and C_28_, corresponding to the high amounts of C_24_, C_26_ and C_28_ fatty acid on *cer*9 mutant leaves (Fig.S1). Around 3.3 to 6.9% (12.2% for *cer*9) of all wax constituents could not be identified.

### Bacterial community analysis by DGGE

**Figure 2:**
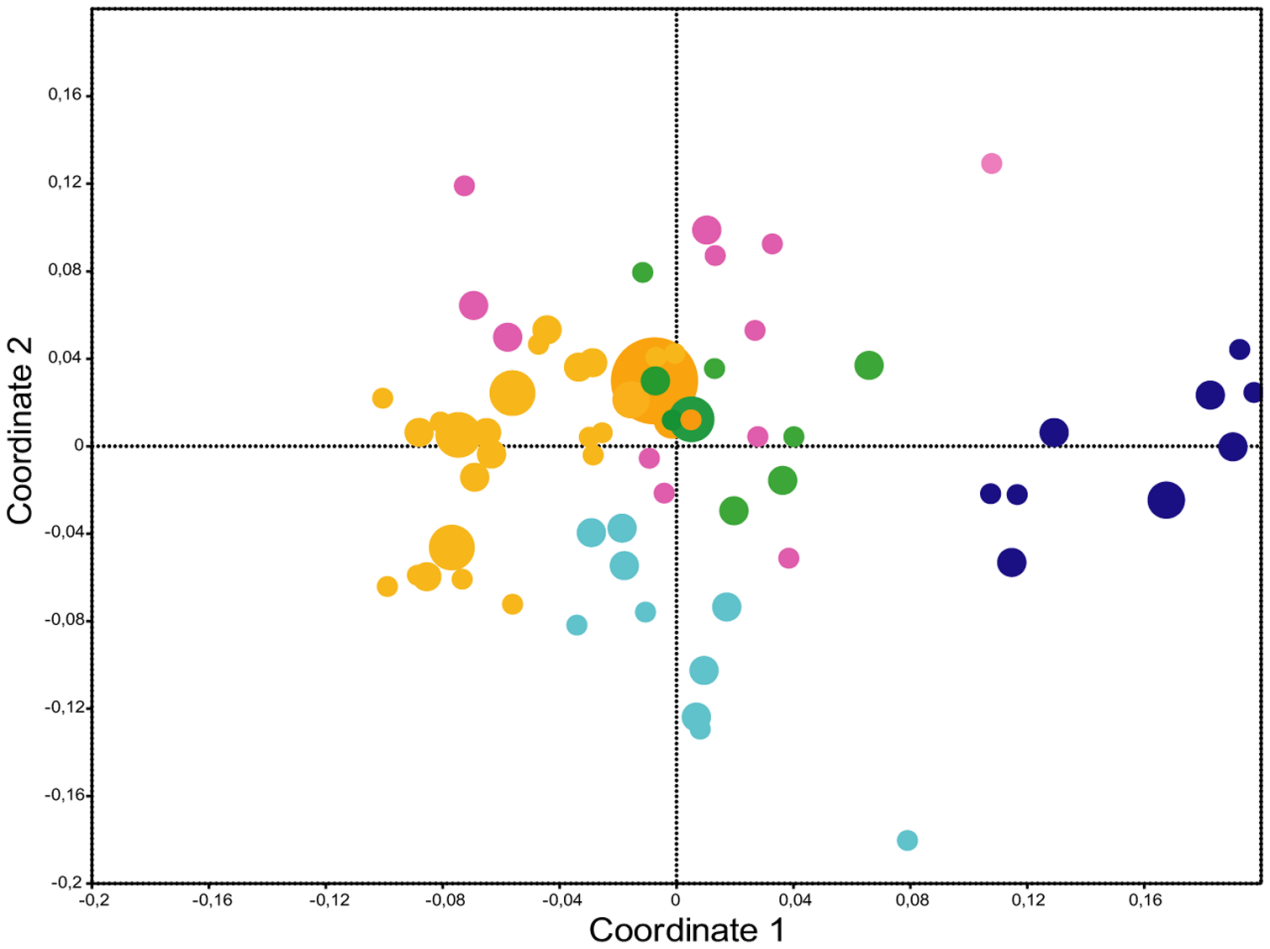
NMDS analysis of DGGE fingerprints. A Nonmetric Multidimensional Scaling plot (stress: 0.1986) of DGGE band presence/absence in technical and biological replicates of bacterial communities derived from the five plant lines is shown. Colour code: yellow: Ler wild type communities, pink: *cer*1 communities, green: *cer*6 communities, dark blue: *cer*9 communities, light blue: *cer*16 communities. Dot size represents number of replicates falling together on the same spot in the plot.

The DGGE experiments were performed in two consecutive years. Dendrograms of the DGGE profiles were created after image analyses based on presence/absence of bands and representative gels are shown in Fig.S2. Although considerable variability between the biological replicates was observed, the dendrograms showed a distinct clustering of the wild type communities compared to the mutant communities. Altogether ten DGGE gels from one year were included in a comparative analysis, each containing wild type and mutant samples on the same gel. A nonmetric multidimensional scaling plot based on the binary information of presence/absence of bands of each community showed that some of the analysed communities, especially *cer*9 and *cer*16, were clearly distinguishable from the other plant lines (Fig.2). An analysis of similarity (ANOSIM, using the Jaccard similarity index) based on the same data matrix showed significant differences between all plant line communities (R = 0.63, pairwise R = 0.3-1, Tab.1).

**Table 1:** Analysis of similarity (ANOSIM) for DGGE banding patterns.

R = 0.63	p = 0.0001			
R	Ler	*cer*1	*cer*6	*cer*9
**Ler**				
***cer*** **1**	0.4772*			
***cer*** **6**	0.2909*	0.1935*		
***cer*** **9**	0.9763*	0.8556*	0.9516*	
***cer*** **16**	0.6183*	0.574*	0.7137*	0.9539*

The analysis was conducted based on a matrix of present/absent band types from ten DGGE gels comparing the bacterial communities derived from the five plant lines. The Jaccard similarity index was used. In line 1 the overall test statistic is given for the comparison of all five groups. Underneath R-values for pairwise comparisons are shown. Asterisks at pairwise comparisons show the significance at the 0.001% level (n = 15–16 for the mutants, n = 56 for the wild type, including technical and biological replicates).

### Bacterial community analysis by amplicon sequencing

For more detailed analysis of the bacterial communities on the five plant lines, three replicate community samples per plant line were subjected to multiplex pyrosequencing. After quality and singleton filtering and removal of sequences affiliated to *A. thaliana* chloroplasts which made up 15.6% of all sequences, 90,815 sequences were recovered from initially 193,289 sequences in total. The smallest sample contained 2,343 sequences (*cer*6 replicate 1) and the largest 10,010 (Ler replicate 2). Altogether 507 OTUs were identified after the clustering into OTUs at the 97% level. Rarefaction curves began to level off at a sampling depth of around 250 sequences. Mean OTU counts ranged between 70 (Ler) and 120 (*cer*9) at a rarefaction value of 2340 sequences (Fig.S3). The richness estimators (Chao1, ACE) indicated that around 85% of the communities had been sampled (Tab.S1). The Pilou evenness estimator and the diversity indices (Shannon, Simpson) indicated relatively uneven communities and rather high diversities in each sample (Tab.S1). The majority of sequences were distributed among only 8 OTUs of the phyla Proteobacteria and Bacteroidetes, which accounted for nearly two thirds of all sequences in the dataset (Tab.S2). The families Sphingomonadaceae (17.6%) and Pseudomonadaceae (18%) as well as an unknown family of the order Sphingomonadales (11%) contained the largest number of sequences. The remaining 499 OTUs (36.74% in total) contained each <2% of the total number of sequences.

**Figure 3:**
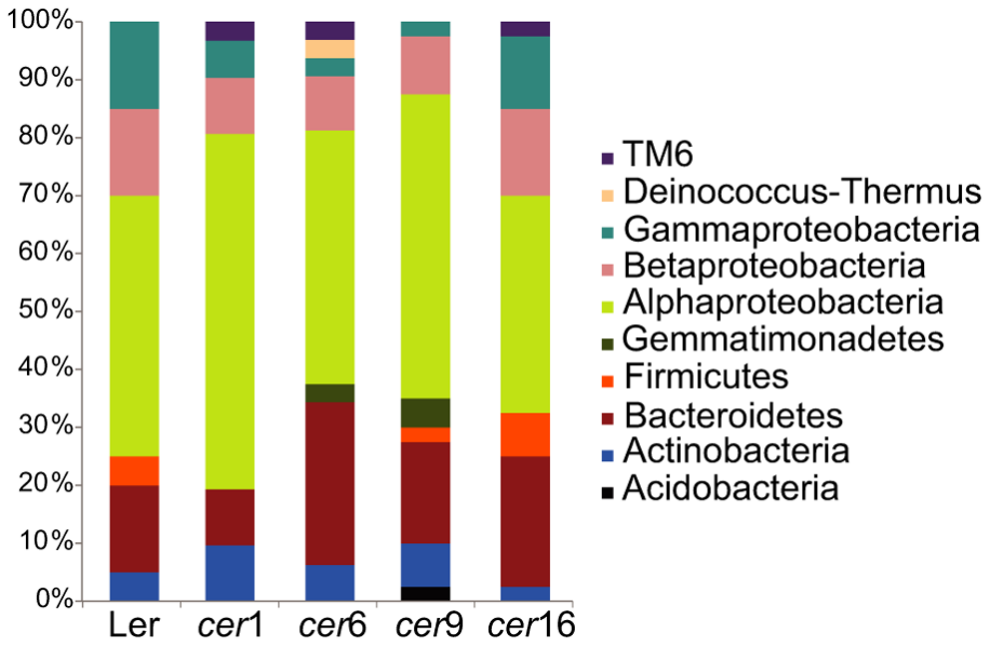
Resident bacterial community diversity of *A. thaliana cer* mutants. The Phylum/subphylum level resolution of the resident microbiota of the five plant lines is shown. The resident community is defined as all OTUs that were present in all three replicate samples of at least one plant line.

Because the read counts between replicates were highly variable, the data were examined based on the presence/absence of the corresponding OTUs. 194 OTUs were found only on an individual replicate in the entire dataset and 234 OTUs were variably present. These OTUs were considered as “transient” members of the Arabidopsis phyllosphere microbiome and no further information could be derived from them (Tab.2). We therefore continued with the “resident” community members of each plant line, referring to those OTUs that were present on all three replicates of at least one plant line (Tab. 2). Altogether 79 of 507 OTUs (15%) satisfied this criterion and all other OTUs were excluded from further analyses. On a phylum level, 56–77% of the resident community OTUs were affiliated with the Proteobacteria, 10–28% with the Bacteroidetes, and 3–10% with Actinobacteria (Fig.3). OTUs belonging to the Firmicutes were only found in the resident communities of Ler, *cer*9 and *cer*16. TM6 phylum OTUs were only found in the resident communities of *cer*1, *cer*6, and *cer*16. A single Deinococcus-Thermus OTU (No. 768) was found on *cer*6 and a single Acidobacteria OTU (No. 173) was found only in the resident community of *cer*9.

**Table 2:** Categories of bacterial phylotypes from the amplicon dataset.

Category	Criteria for definition	No. of OTUs in this category (non-rarified/rarefied)	Percentage of total sequence count
Transient community	OTUs that were absent from at least one replicate of a given plant line	431/387	15.7%
Resident community	OTUs that were present in all three replicates of a given plant line	79/76	84.3%
Core community	OTUs of the resident community, that were present in all or all but one plant line communities	13/13	56.7%
Plant line-specific community	OTUs of the resident community that were present in one plant line, but were absent from at least one other plant line	35/23	3.5%
Plant line-unique community	OTUs that were uniquely present in the resident community of one plant line	20	
	OTUs that were uniquely absent in the resident community of one plant line	10	

The criteria for the different categories are explained. Three replicates for each plant line (*A. thaliana* Ler, *cer*1, *cer*6, *cer*9, *cer*16) were analyzed. Percentages of total sequence count are derived from rarefied datasets. No percentage values are given for the plant line-unique communities, as they together form the plant line-specific community.

**Figure 4:**
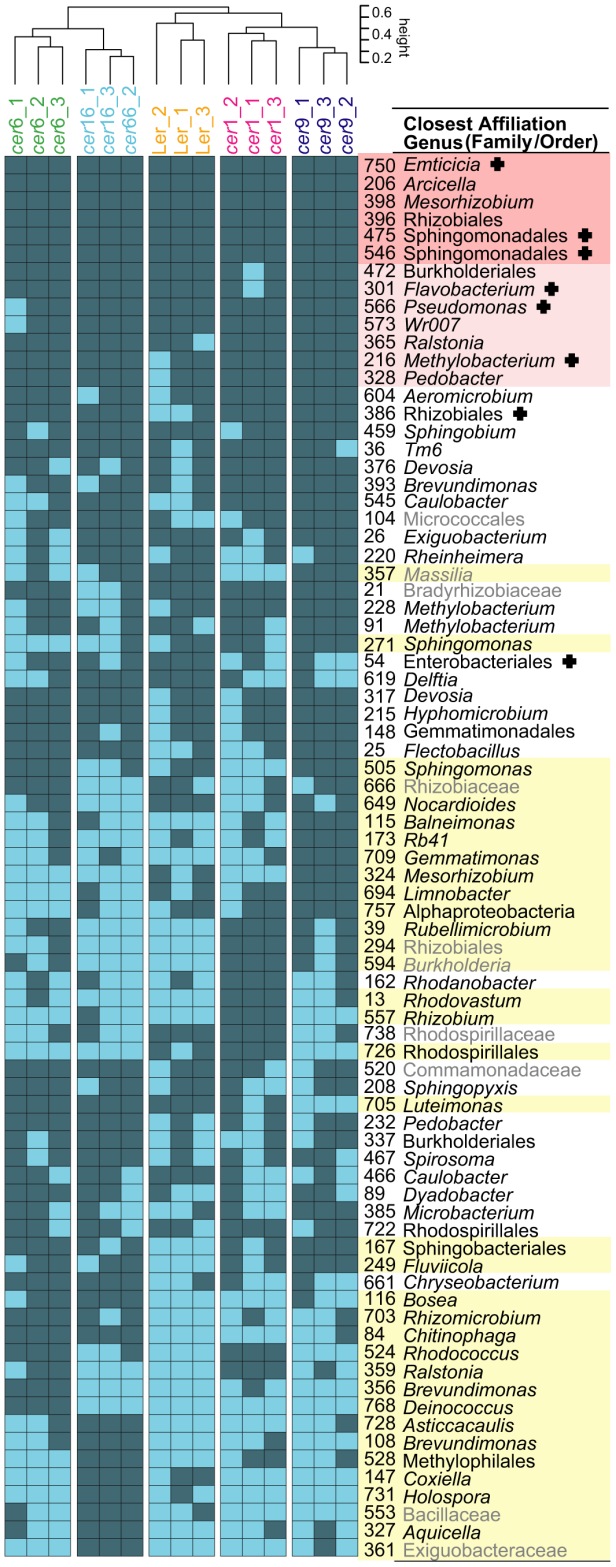
OTU level resolution of plant line resident bacterial communities. A heatmap of the resident community OTUs of the five plant lines under investigation (present = dark blue, absent = light blue) is shown. The closest possible taxonomic affiliation is given. Grey writing indicates that these taxa were identified as chimeras in either the Greengenes or the Silva_r108 reference dataset but not in both. The six ubiquitously present OTUs are highlighted in dark red and the OTUs that are present in 14/15 samples are marked in light red (“core” community). All taxa that were found in at least one plant line and were absent from at least one other plant line are highlighted in yellow (“plant line-specific” community). OTUs marked with “+” also contained highest sequence counts (Tab.S3).

Of the 79 OTUs forming the resident communities, six were present in all sampled communities (highlighted in red) and seven additional OTUs were present in all but one replicate (highlighted in light red), (Fig.4). These 13 nearly ubiquitously present OTUs were considered as the resident “core” community (Tab.2). Among the ubiquitous OTUs were also several that contained highest sequence read counts (indicated by a cross, Fig.4, Tab.S2). 35 OTUs were differentially present on the plant lines under investigation which means that they were present in all replicates of at least one plant line, but were absent in all replicates of at least one other plant line (highlighted in yellow). These OTUs were defined as the “plant line-specific” community (Tab.2). A dendrogram based on similarities showed a coherent clustering of the replicates among the five plant line resident communities (Fig.4 top).

**Figure 5:**
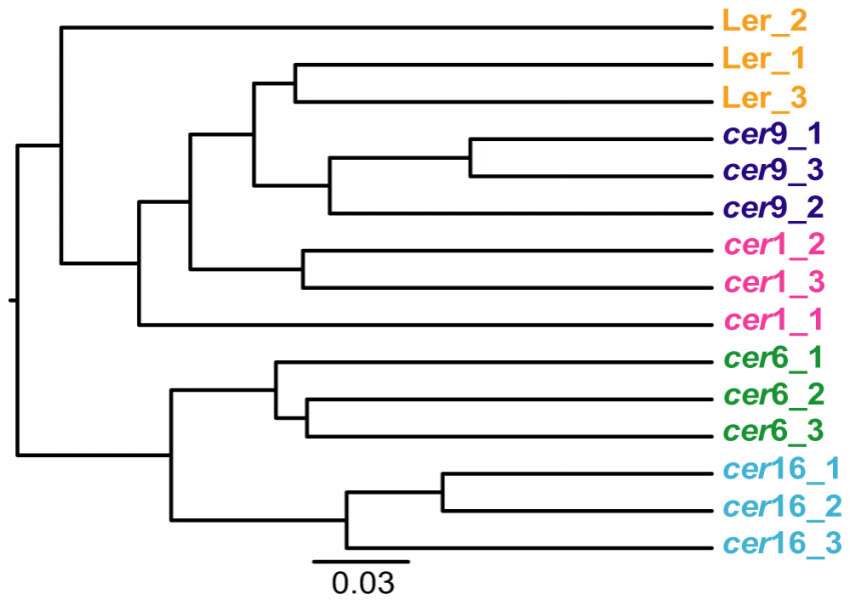
UniFrac distance based hierarchical clustering (UPGMA) of the resident microbiota. The resident community data was extracted from a rarified dataset based on Greengenes phylogeny assignment.

Further analyses were carried out after extraction of the 76 resident community OTUs from a rarefied dataset, excluding all chimeras detected with the Greengenes dataset. Rarification was performed here in order to avoid biases resulting from different sample sizes during statistical analyses. A hierarchical clustering of the resident communities based on unweighted UniFrac distances grouped the replicate plant line communities of *cer*6, *cer*9 and *cer*16 together (Fig.5). The replicate Ler_2 was distant from the other two Ler replicates which is consistent with the lower diversity of this sample. Further, replicate *cer*1_1 did not group coherently with the other two *cer*1 replicates. An NMDS plot based on similarities between the resident communities of the five plant lines confirmed largely the clustering of replicates (Fig.S4). An ANOSIM of these data revealed significant differences between several analysed resident communities (Tab.3). Due to the limited sample size, pairwise comparisons could only be made on a significance level of 0.1%. Under these circumstances the resident communities of Ler/*cer*6, *cer*1/*cer*6, *cer*6/*cer*9, *cer*6/*cer*16, and *cer*9/*cer*16 were found to be significantly different. Further analyses in support of our results are provided as supporting information (Analysis.S1&S2). A Mantel test showed that the resident communities of each of the five plant lines were significantly correlated (p = 0.0054) with the wax profiles (R = 0.2618).

**Table 3:** Analysis of similarity for bacterial resident communities in the phyllosphere of *A. thaliana* Ler wild type and four *cer* mutant lines as analysed by amplicon pyrosequencing.

R = 0.6615	p = 0.0001			
R	Ler	*cer*1	*cer*6	*cer*9
**Ler**				
***cer*** **1**	0.6296			
***cer*** **6**	0.6667*	0.7593*		
***cer*** **9**	0.5185	0.7037	0.6296*	
***cer*** **16**	0.6296	0.963	0.8148*	1*

The ANOSIM analysis, conducted using the Jaccard similarity index, is based on a binary resident community dataset extracted from a rarified OTU table. In line 1 the overall test statistic is given for the comparison of all five groups. Underneath R-values for pairwise comparisons are shown. Asterisks at pairwise comparisons show the significance at the 0.1% level (lower significance levels could not be tested due to the small sample size (n = 3 for each group)).

**Figure 6:**
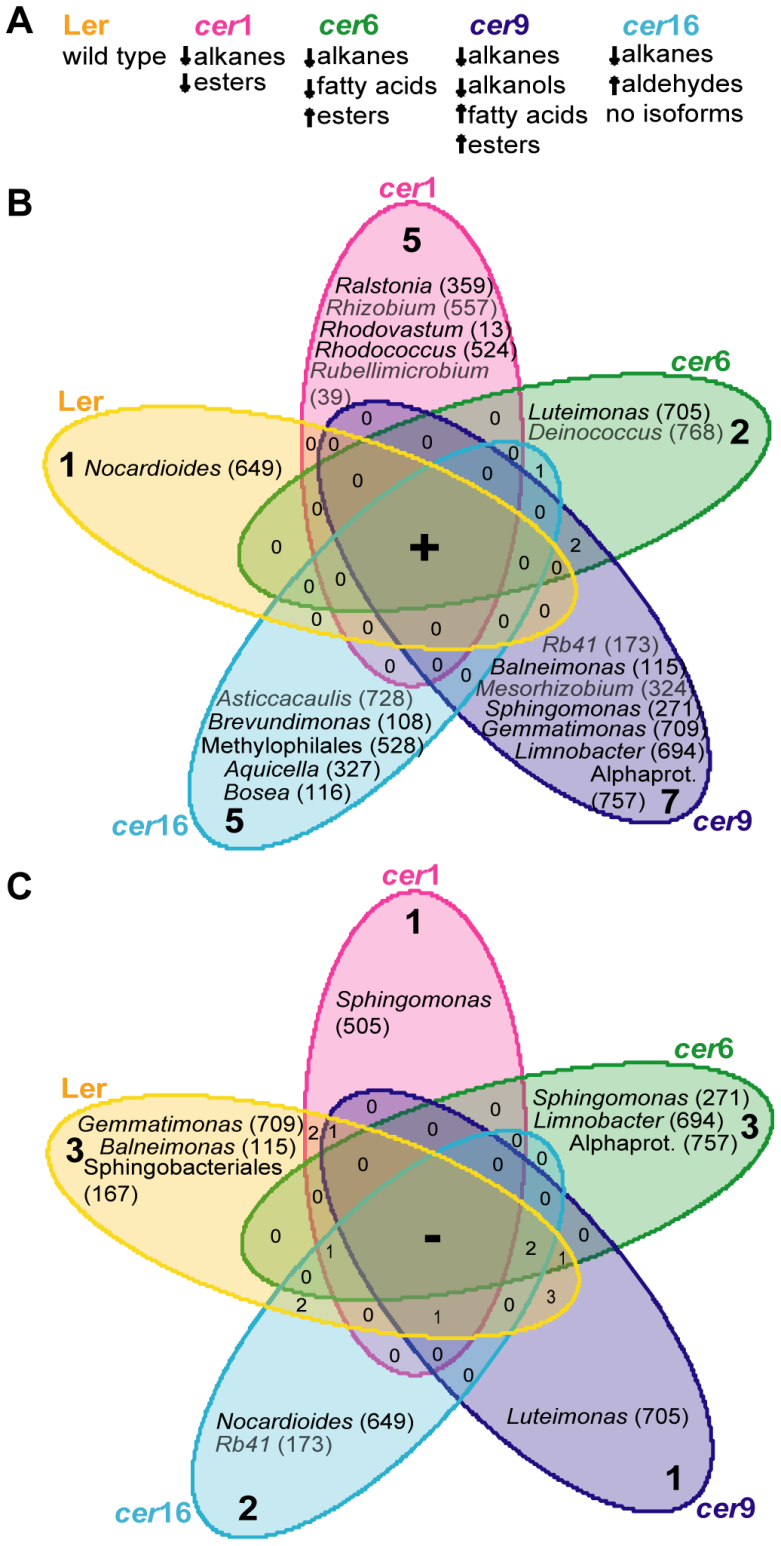
Venn diagram of the plant-line specific communities. Counts of uniquely present (Fig. 6A) or uniquely absent (Fig. 6B) phylotypes are shown. OTUs that were present or absent on multiple plant lines are indicated by numbers in the overlapping areas of the diagram. Grey writing indicates phylotypes, which were represented by sequence abundances below 1% of the plant line-specific communities. The plant line-specific community data was extracted from a rarified dataset and only those OTUs were included that were found non–chimeric with both (Greengenes and Silva_108) reference datasets. The closest possible taxonomic affiliation is given. OTU numbers are given in brackets.

We further elaborated the analysis of the “plant line-specific” community based on a rarefied dataset. This effort was undertaken in order to highlight the “plant line-specific” OTUs, which contributed most to the statistical differences between plant lines under the most stringent selection criteria (Tab.2). In Figure 6 we show those OTUs that are uniquely present (Fig.6B) or uniquely absent (Fig.6C) in the resident community of a given plant line as well as the respective major wax compositional changes (Fig.6A). In doing so, 5, 2, 7, and 5 uniquely present OTUs were identified in the resident communities of the mutants *cer*1, *cer*6, cer9, and *cer*16. When analysed for the absence of OTUs, the mutants were specifically missing 1 (*cer*1), 3 (*cer*6), 1 (*cer*9), and 2 (*cer*16) OTUs in comparison to the other plant lines.

## Discussion

### Changes in *A. thaliana* leaf wax composition following outdoor growth

Our analysis of the cuticular waxes of the *cer*1, *cer*6, *cer*9 and *cer*16 mutant leaves confirmed previously described alterations in wax coverage and composition of these mutants compared to the Ler wild type [23]. However, the investigated plant lines grown under outdoor conditions exhibited an about two- to fourfold increase in wax coverage over cabinet grown Landsberg *erecta* [38], [39]. It appears thus likely that our findings reflect an adaption of the plants to the outdoor environment [40]. Effects of environmental variables, such as UV radiation, humidity, temperature, and water availability on the cuticular wax composition were previously reported [11], [41]. Specifically, quantitative changes in e.g. the ester fraction of outdoor grown *Brassica* species as well as during development of *A. thaliana* are described in the literature [42], [12]. Such environmentally or developmentally induced shifts in wax composition might explain our finding of considerable amounts of alkyl esters on the *cer*6 and *cer*9 mutants (Fig.1). To our knowledge the presence of monoglycerides of fatty acids, which are known from *A. thaliana* root waxes, has not yet been reported on leaf waxes of *A. thaliana* [43]. Moreover the absence of branched chain isoforms of alkanols and other components in the *cer*16 mutant is a novel observation. Taken together, we observed a chemically distinct cuticular wax composition on the five investigated plant lines. In agreement to data obtained from six week old, cabinet grown plants [23], we could confirm the consistency of the mutation-induced phenotypical changes after the 13-weeks partially outdoor-growth period.

### Microbial community analysis by DGGE

DGGE analysis conveys an overview of the major members of microbial communities [44]. Thus we used it as a first screening tool for the analyses of the communities of the four *cer* mutant lines and their respective wild type. During two subsequent years the analyses of three to four wax mutant lines in comparison to the wild type clearly showed distinct bacterial community patterns (Fig.S2). The NMDS plot of the community patterns in one year revealed some clusters which appeared to be overlapping whereas others clustered separately (Fig.2). The statistical evaluation of these DGGE banding patterns confirmed significantly different bacterial communities in the phyllosphere of the different plant lines.

### Resident vs. transient microbial communities

The investigation of the leaf associated microbiota of the five plant lines using amplicon pyrosequencing revealed uneven and diverse bacterial communities, as judged from population statistical analyses (Tab.3). In terms of abundance, eight OTUs within the Proteobacteria and Bacteroidetes were detected that comprised nearly two thirds of the total sequence count (Tab.S2). This weighting might be due to primer bias [45]. However, these OTUs were frequent members of the resident community when analyzed for presence/absence and are thus likely to be common members of the community. These OTUs were affiliated with the Flavobacteriaceae, Flexibacteriaceae, Methylobacteriaceae, Rhizobiaceae, Sphingomonadaceae, Enterobacteriaceae and Pseudomonadaceae. Members of most of these bacterial families were found earlier in *A. thaliana* phyllosphere communities from natural stands in Spain [46] and from Michigan, USA [47]. This pattern supports the existence of a permanent phyllosphere microbiome associated with *A. thaliana* which is independent of its geographic location.

In spite of this, a sizable transient microbial community was observed, amounting to 431 of 507 OTUs in total. Variability was not only observed with respect to read counts but also between replicate samples. We cannot completely exclude contaminations with plant endophytes or variations, which might have occurred during the harvesting process of the bacteria, although the kind of technique is commonly used [47], [48]. It is however very unlikely, that biases, resulting from different adhesion possibilities for bacteria on the leaves of the different plant lines occurred as *A. thaliana* generally exhibits a smooth wax layer on its leaf surfaces [49]. Examinations of the investigated plant lines grown in controlled climate conditions using SEM did not show structural alterations between the mutants and the wild type surfaces (data not shown). Plant leaves are commonly perceived as a heterogeneous habitat for microorganisms that is heavily influenced by environmental conditions [50]–[53]. Thus, bacterial colonization under natural conditions occurs likely with a high degree of variability [54], [48]. To account for this variation we relied on replicate sampling [55]. We focused our further analysis on the resident microbial community (Tab.2). Furthermore, the dataset was analysed with regard to presence/absence of OTUs only. In doing so, we could confirm a distinct clustering between the microbial communities and the different *cer* mutants (Fig.5, Fig.S4). Several pairwise comparisons between plant lines and resident microbiota were confirmed to be significantly different, although a greater number of replicates would have made the analyses more comprehensive (Tab.3). The observations were supported by bacterial community structure analyses in two subsequent years using DGGE-fingerprinting (Fig.S2) and further analyses of the amplicon dataset (Analysis.S1&2).

### Arabidopsis core vs “plant line-specific” communities

The resident microbiota which comprised over 84% of the total sequence count was further divided into a core community and a “plant line-specific” community (Fig.4, Tab.2). The bacterial core community comprises phylotypes present on all or nearly all replicate communities of all plant lines. This part of the community is apparently not influenced by the wax phenotype, or at least an influence could not be detected. Some of the phylotypes, like Sphingomonadales, *Pseudomonas* and Burkholderiales, might be general phyllosphere colonizers, as they have been detected on many other plant species as well [1], [8]. However, other phylotypes of the core community, like *Pedobacter* or *Flavobacterium*, seem to be more specific to *A. thaliana* [1], [47]. Interestingly, a number of these genera were shown to be seed transmitted [56] which suggests their permanent association with *Arabidopsis* plants. In summary, while it remains unknown exactly how these core community members arrive at the leaf surface, the effects of wax chemistry appear to play a minor role for this process.

Figure 6 depicts a subset of “plant line-specific” OTUs that were uniquely present or uniquely absent on a given plant line. With this even more stringent criterion we sought to identify those OTUs that are most specific for a given plant phenotype. A dataset rarefied to the smallest sampling effort was used here to assure for the most stringent observation of the data. Further, phylotypes present at low sequence numbers are labelled accordingly. A number of OTUs were identified with this approach, the majority of which are known as typical plant phyllosphere inhabitants. Bacteria belonging to the genera *Sphingomonas* and *Rhizobium* are known as plant colonizers [1], [8], [56], [57]. Also *Nocardioides*, *Rubellimicrobium*, *Rhodococcus*, *Ralstonia*, *Deinococcus*, *Balneimonas* and *Mesorhizobium* have been found in association with soybean, rice, clover, *Arabidopsis*, *Tamarix* or spinach [1], [57], [58]. Several phylotypes (*Ralstonia*, *Rhodococcus*, *Deinococcus*, *Bosea*, *Rhodovastum*) are described as biodegraders, extremophiles or photoorganotrophs [59]–[63]. Such metabolic properties would be consistent with a life style on leaf surfaces, being perceived as an extreme, heterogeneous habitat [1], [50], [64].

Following outdoor growth, the wild type *A. thaliana* resident community was found to be specifically enriched by only a single bacterial clade (OTU 649), whereas the *cer* mutants acquired specifically as many as 2–7 bacterial clades (Fig.6). This observation suggests that the cuticle of wild type *A. thaliana* is less readily colonized by bacteria than the cuticles of the investigated wax mutants, which are apparently more prone to bacterial immigration. This hypothesis is consistent with earlier observations showing that bacterial populations on wild type cuticular waxes are reduced in comparison to those of wax mutants in maize [21]. However, direct effects of cuticular components on specific bacterial strains remain to be demonstrated.

In the present study a Mantel test comparing the resident communities with the cuticular wax composition of the respective plants showed that these parameters were correlated. There are numerous hypotheses as to how surface wax composition could affect phyllosphere microbial diversity [65]. Alterations in wax composition might affect wettability of the leaves, manipulating thus bacterial biosurfactant action, adhesion possibilities, quorum sensing signalling and/or water availability for the cells [51], [66]–[69]. Nutrient leaching from the plant can be affected directly or by altered diffusion possibilities of bacterial effectors through the cuticle [70]–[73]. Moreover, an altered cuticular wax composition might have direct effects on the microbiota, either by acting as an antimicrobial agent or by providing a carbon source for bacteria [74]–[76]. Thus, cuticle properties might create niches for specifically adapted bacteria. These specificities in microbial community composition could conceivably induce shifts on other bacterial players in a synergistic (i.e. nutritional interdependencies) or antagonistic (i.e. antimicrobial compounds) manner [54], [77], [78]. For example, it has been shown that pre-inoculation of *Citrus* and tomato plants with plant growth promoting and biocontrol bacteria like *Azospirillum brasiliense* or *Bacillus* strains, which themselves have no negative effects on the plants, prevents the development of large populations of pathogenic bacteria [79], [80]. For *Arabidopsis*, antagonistic effects of specifically plant-associated *Sphingomonas* strains to a plant-pathogenic *Pseudomonas syringae* strain were described [81]. Thus it might be conceivable, that plant cuticular properties foster the growth of natural commensal microbiota that may ward off incoming pathogens as discussed by Vorholt [1]. Mechanistic studies, using selected bacterial isolates from the phyllosphere in combination with specifically mutated plants will aid in deciphering the interactions between the plant cuticle and environmental bacteria in more detail.

### Concluding remarks

The present study shows that *A. thaliana* plant lines with altered cuticular lipid biosynthesis harbour distinct phyllosphere bacterial communities. Our findings are based on the analysis of four different *cer* mutant lines and the Ler wild type and are supported by two cultivation independent methodologies (DGGE, amplicon pyrosequencing). Amplicon sequencing revealed both, transient and resident members of the leaf microbiota. Moreover, within the resident community, this technique allowed for the identification of core and plant line-specific bacterial clades. To our knowledge this is the first report on the effects of cuticular wax composition on bacterial leaf colonisation under natural conditions. Our findings suggest that plant wax chemistry might indeed serve as a selection factor that influences the microbial community composition in the phyllosphere.

## Supporting Information

Figure S1 **Cuticular wax analysis of the five** ***A. thaliana*** **lines grown partially under outdoor growth conditions.** Chain length distributions of the main compound classes are shown. Abbreviations: a.c.  =  additional components (combining sterols, triterpenoid-like compounds, C_29_ ketone, secondary alkanols and monoglycerides of fatty acids in *cer*9), n.i.  =  not identified compounds.
(PDF)

Figure S2 **Representative DGGE gels of 2010 (A) and 2011 (B) sampled bacterial communities.** QuantityOne® image analyses (UPGMA) dendrograms are depicted below the gels.
(PDF)

Figure S3 **Rarefaction curves of the amplicon pyrosequenced community samples.** The rarefaction curves show the observed number of OTUs with increasing number of subsampled sequences. Mean rarefaction curves with deviation for the five plant line communities are shown. Color code: yellow: Ler wild type; pink: *cer*1; green: *cer*6; dark blue: *cer*9; light blue: *cer*16.
(PDF)

Figure S4 **NMDS analysis of the bacterial resident communities derived from the amplicon dataset.** An NMDS plot (stress: 0.1997) based on the presence/absence of OTUs in the five bacterial resident communities using the Jaccard similarity index is given. The resident community data was extracted from a rarified dataset. Color code: orange: Ler wild type; pink: *cer*1; green: *cer*6; dark blue: *cer*9; light blue: *cer*16.
(PDF)

Table S1 Evenness and richness estimators and diversity indices of leaf phyllosphere bacterial communities of three replicate samples from *A. thaliana* wild type and four *cer* mutant lines as analysed by amplicon pyrosequencing.
(DOCX)

Table S2 Most abundant OTUs as defined on read count from *A. thaliana* leaf phyllosphere derived bacterial communities as analysed by amplicon pyrosequencing.
(DOCX)

Analysis S1 **Additional NMDS and ANOSIM Analyses of the amplicon dataset.** Analysis of all OTUs which were present in more than one replicate of at least one plant line.
(PDF)

Analysis S2 **Additional NMDS and ANOSIM Analyses of the amplicon dataset.** Analysis of all OTUs which were present in more than one replicate of the dataset, regardless of the plant line.
(PDF)

Sequence File S1 **Denoised sequences.**
(ZIP)

Sequence File S2 **OTUs and respective sequence IDs.**
(TXT)

Sequence File S3 **Representative sequences.**
(FNA)

Sequence File S4 **Aligned representative sequences.**
(FASTA)

Sequence File S5 **OTU-table.**
(TXT)
