# Supplementary figures and images for: Distinct Phyllosphere Bacterial Communities on Arabidopsis Wax Mutant Leaves
Source: PLoS One. 2013 Nov 5;8(11):e78613. doi: 10.1371/journal.pone.0078613 (PMC3818481; doi:10.1371/journal.pone.0078613)

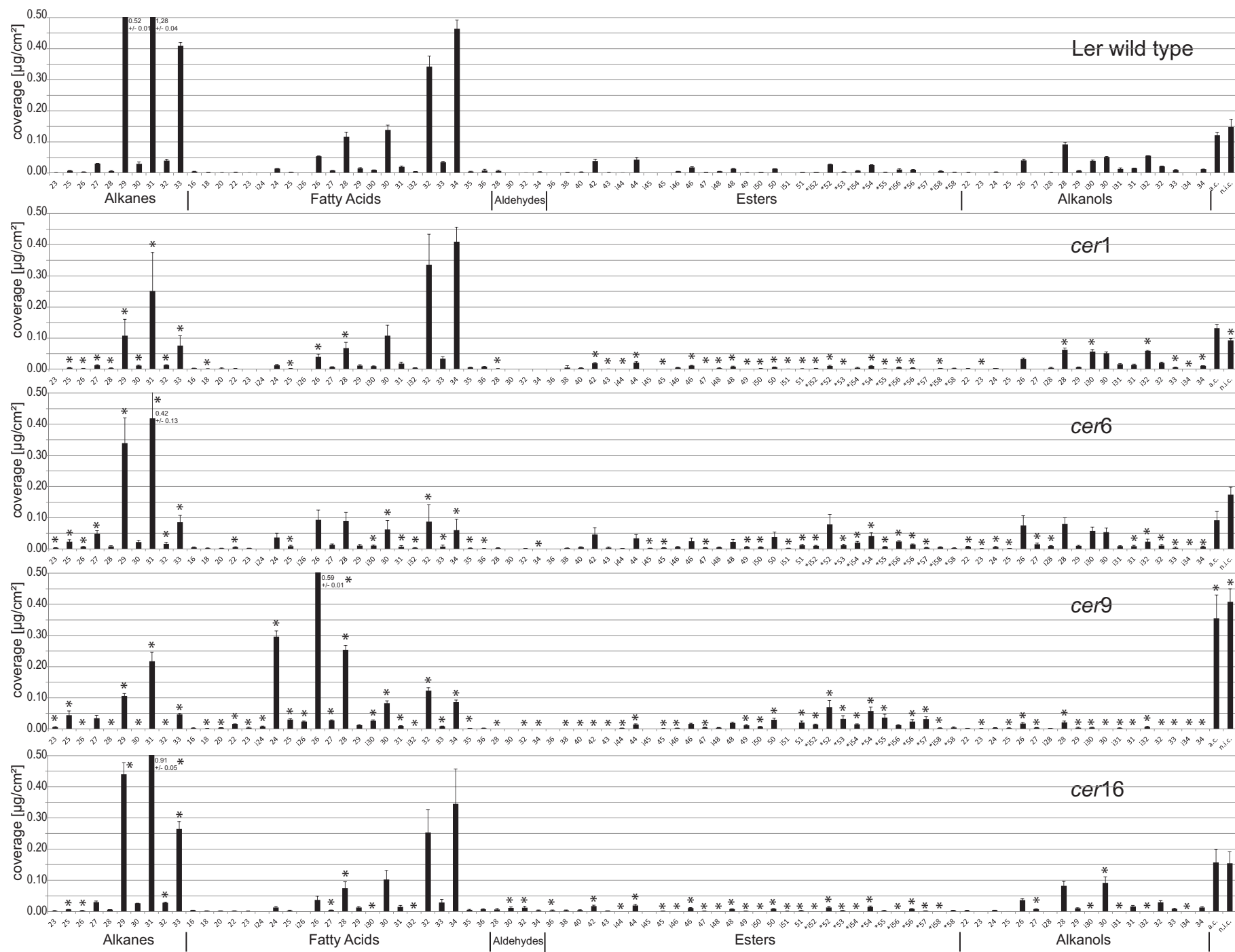

Supplement: Figure S1 — Cuticular wax analysis of the five A. thaliana lines grown partially under outdoor growth conditions. Chain length distributions of the main compound classes are shown. Abbreviations: a.c. = additional components (combining sterols, triterpenoid-like compounds, C29 ketone, secondary alkanols and monoglycerides of fatty acids in cer9), n.i. = not identified compounds. (PDF) [file pone.0078613.s001.pdf]

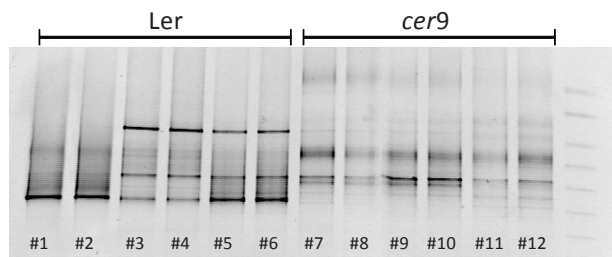

0.44 0.60 1.00

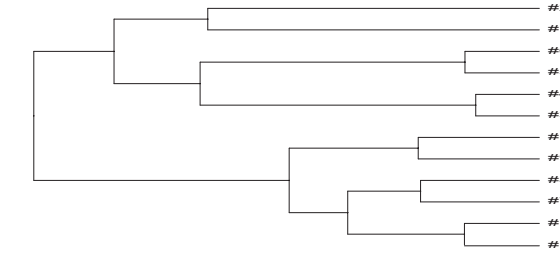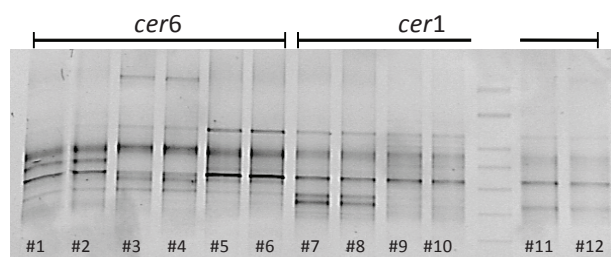

0.49 0.60 0.70 0.80 0.90 1.00

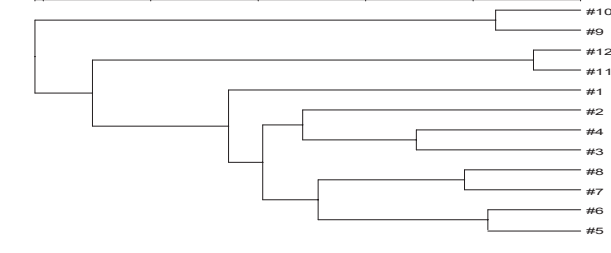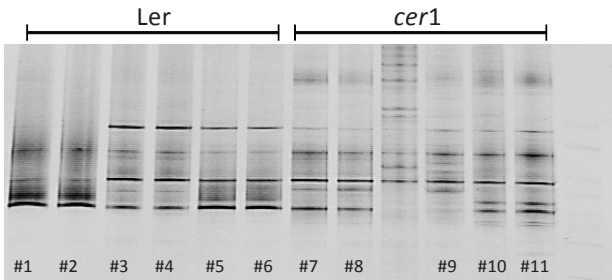

0.53 0.70 0.80 1.00

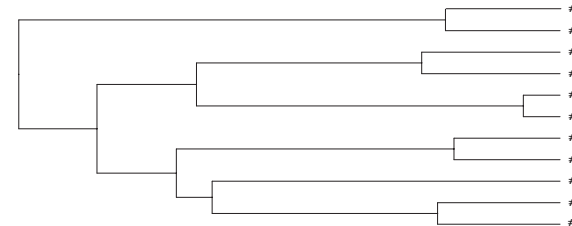

A

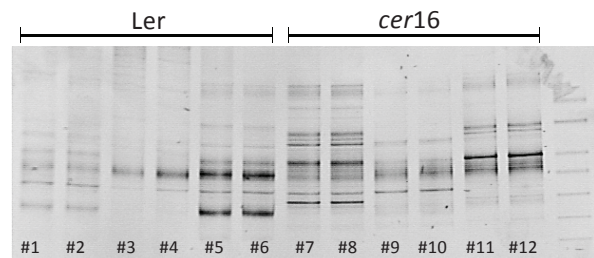

0.48 0.60 0.70 0.80 0.90 1.00

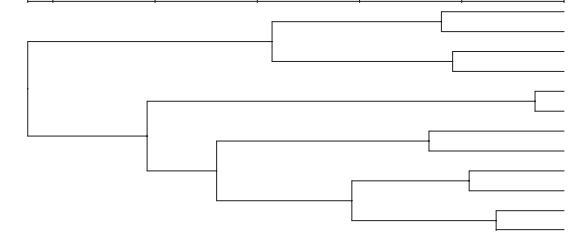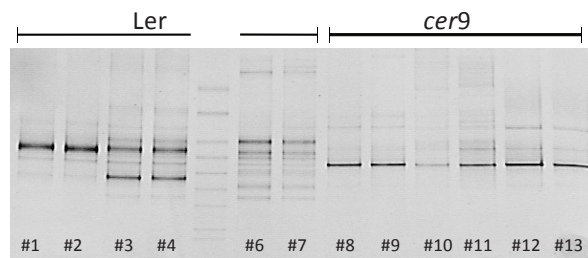

0.24 0.40 0.60 0.80 1.00

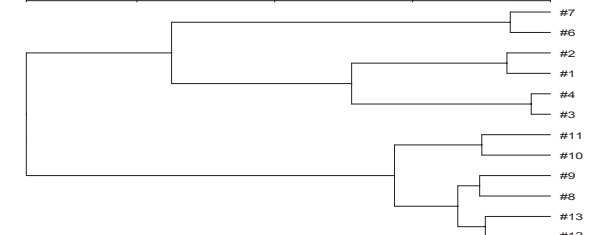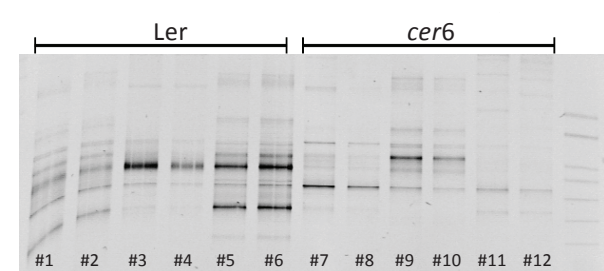

0.44 0.60 1.00

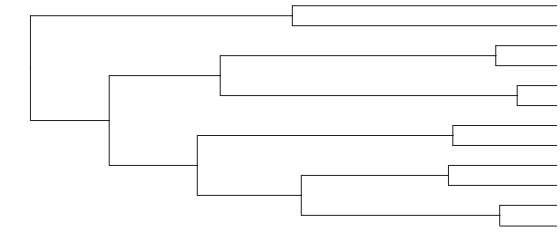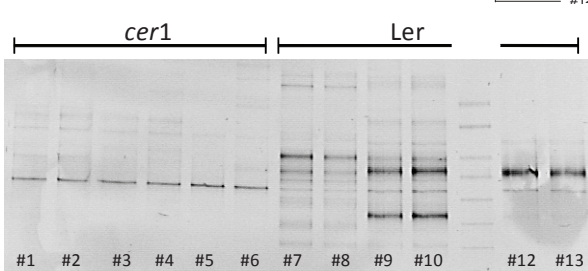

0.44 0.60 0.70 0.80 0.90 1.00

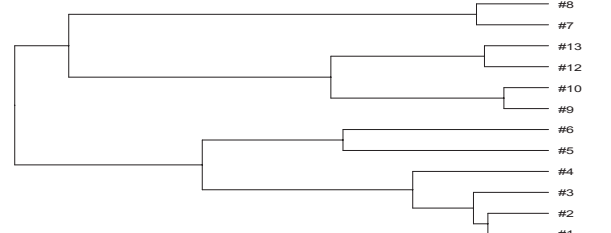

B

Supplement: Figure S2 — Representative DGGE gels of 2010 (A) and 2011 (B) sampled bacterial communities. QuantityOne® image analyses (UPGMA) dendrograms are depicted below the gels. (PDF) [file pone.0078613.s002.pdf]

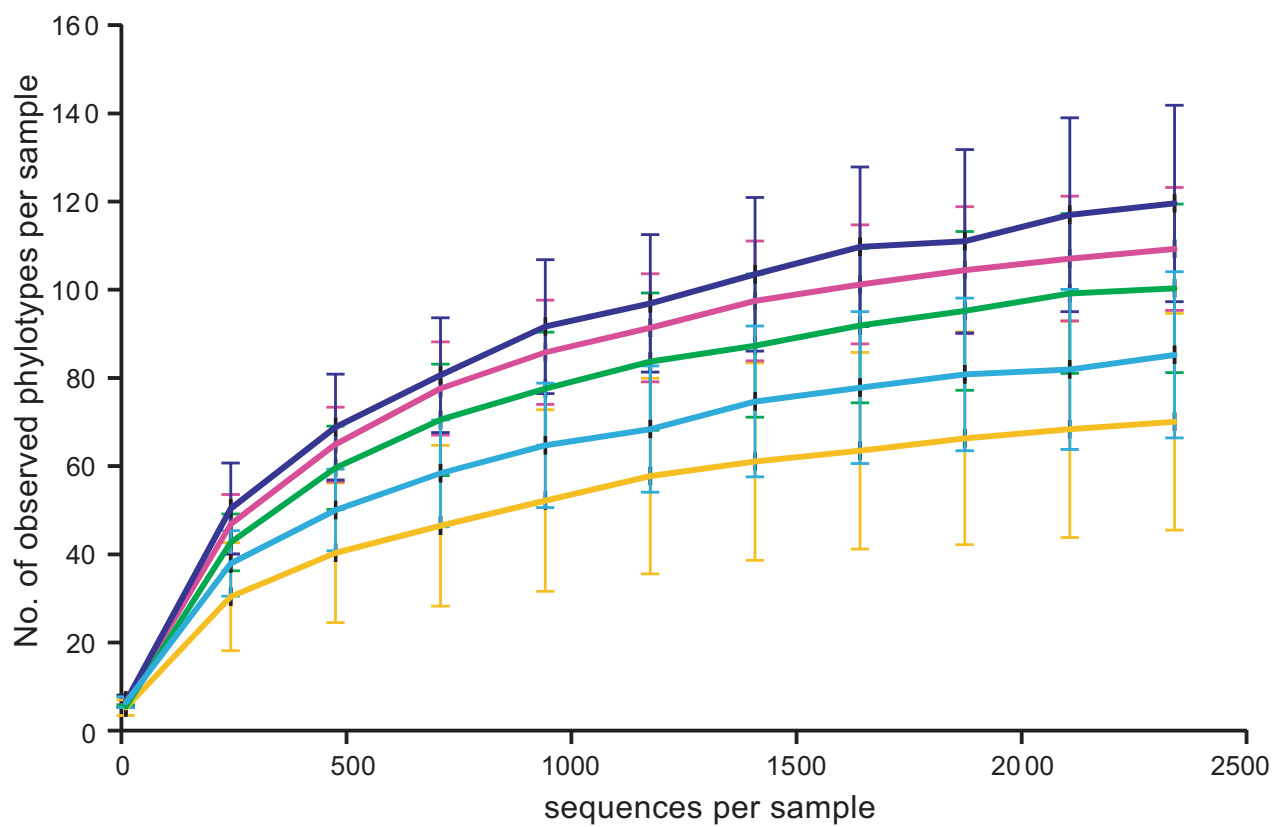

Supplement: Figure S3 — Rarefaction curves of the amplicon pyrosequenced community samples. The rarefaction curves show the observed number of OTUs with increasing number of subsampled sequences. Mean rarefaction curves with deviation for the five plant line communities are shown. Color code: yellow: Ler wild type; pink: cer1; green: cer6; dark blue: cer9; light blue: cer16. (PDF) [file pone.0078613.s003.pdf]

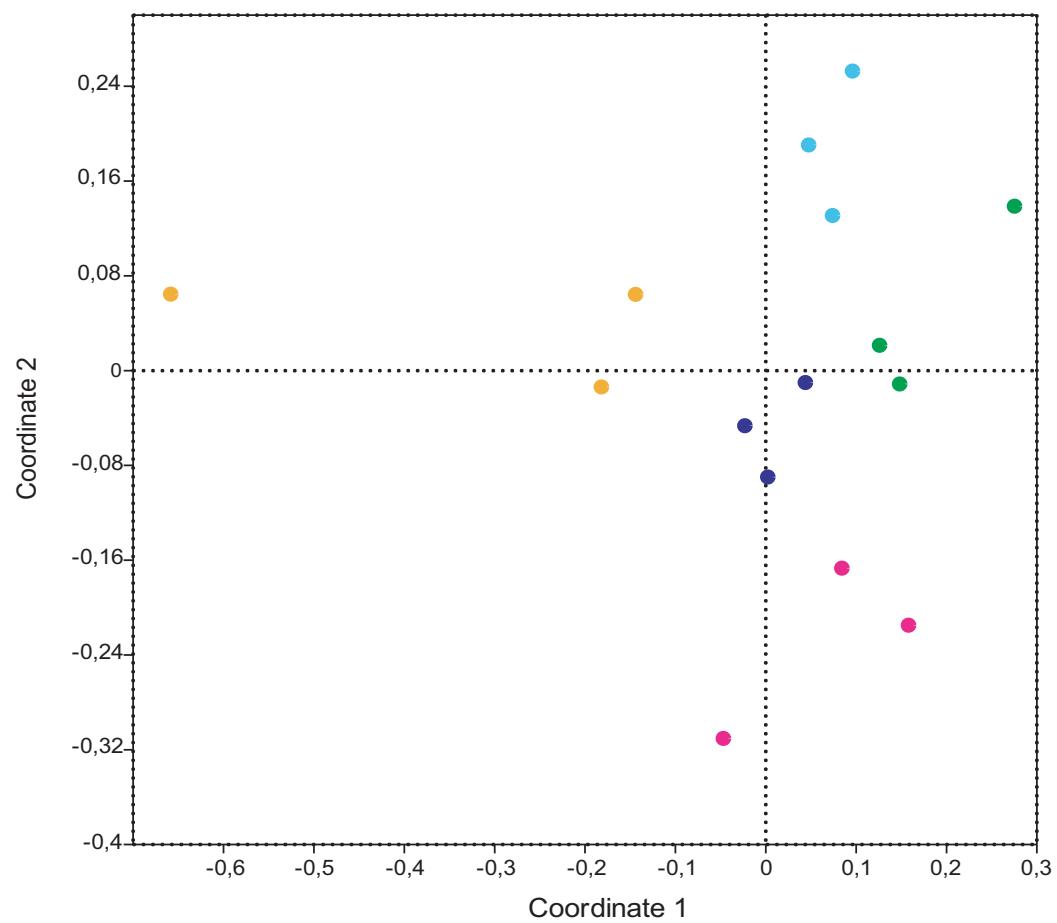

Supplement: Figure S4 — NMDS analysis of the bacterial resident communities derived from the amplicon dataset. An NMDS plot (stress: 0.1997) based on the presence/absence of OTUs in the five bacterial resident communities using the Jaccard similarity index is given. The resident community data was extracted from a rarified dataset. Color code: orange: Ler wild type; pink: cer1; green: cer6; dark blue: cer9; light blue: cer16. (PDF) [file pone.0078613.s004.pdf]
