# Supplementary material for: Distinct Phyllosphere Bacterial Communities on Arabidopsis Wax Mutant Leaves
Source: PLoS One. 2013 Nov 5;8(11):e78613. doi: 10.1371/journal.pone.0078613 (PMC3818481; doi:10.1371/journal.pone.0078613)
Supplement: Table S1 — Evenness and richness estimators and diversity indices of leaf phyllosphere bacterial communities of three replicate samples from A. thaliana wild type and four cer mutant lines as analysed by amplicon pyrosequencing. (DOCX) [file pone.0078613.s005.docx]

**Table S1**: **Evenness and richness estimators and diversity indices of leaf phyllosphere bacterial communities of three replicate samples from *A. thaliana* wild type and four *cer* mutant lines as analysed by amplicon pyrosequencing.**

|  |  | **Richness** | **Evenness** | **Diversity** |  |  |
| --- | --- | --- | --- | --- | --- | --- |
|  | **OTU Count** | **Chao1** | **ACE** | **Pilou** | **Shannon** | **Simpson** |
| Ler1 | 93 | 104 ± 7 | 106 ± 5 | 0.63 | 2.86 | 0.86 |
| Ler2 | 35 | 38 ± 3 | 40 ± 3 | 0.27 | 0.94 | 0.39 |
| Ler3 | 76 | 89 ± 9 | 91 ± 5 | 0.65 | 2.83 | 0.91 |
| *cer*1_1 | 116 | 120 ± 3 | 122 ± 5 | 0.54 | 2.58 | 0.75 |
| *cer*1_2 | 87 | 106 ± 12 | 105 ± 5 | 0.63 | 2.80 | 0.89 |
| *cer*1_3 | 122 | 149 ± 14 | 145 ± 6 | 0.71 | 3.43 | 0.94 |
| *cer*6_1 | 82 | 97 ± 11 | 95 ± 5 | 0.63 | 2.76 | 0.86 |
| *cer*6_2 | 96 | 114± 11 | 113 ± 5 | 0.53 | 2.41 | 0.79 |
| *cer*6_3 | 124 | 146 ± 11 | 148 ± 6 | 0.61 | 2.94 | 0.82 |
| *cer*9_1 | 110 | 151 ± 24 | 131 ± 5 | 0.63 | 2.95 | 0.88 |
| *cer*9_2 | 151 | 180 ± 13 | 189 ± 7 | 0.75 | 3.75 | 0.95 |
| *cer*9_3 | 103 | 116 ± 7 | 123 ± 5 | 0.56 | 2.60 | 0.82 |
| *cer*16_1 | 111 | 125 ± 8 | 130 ± 5 | 0.68 | 3.19 | 0.91 |
| *cer*16_2 | 78 | 95 ± 12 | 94 ± 5 | 0.60 | 2.59 | 0.85 |
| *cer*16_3 | 65 | 82 ± 15 | 78 ± 4 | 0.60 | 2.49 | 0.86 |
