# Supplementary material for: Distinct Phyllosphere Bacterial Communities on Arabidopsis Wax Mutant Leaves
Source: PLoS One. 2013 Nov 5;8(11):e78613. doi: 10.1371/journal.pone.0078613 (PMC3818481; doi:10.1371/journal.pone.0078613)
Supplement: Table S2 — Most abundant OTUs as defined on read count from A. thaliana leaf phyllosphere derived bacterial communities as analysed by amplicon pyrosequencing. (DOCX) [file pone.0078613.s006.docx]

**Table S2:** **Most abundant OTUs as defined on read count from *A. thaliana* leaf phyllosphere derived bacterial communities as analysed by amplicon pyrosequencing.**

| **OTU No.** | **Sequence Count** | **% of total Sequence Count** | **Affiliation**  **Phylum** | **Class** | **Order** | **Family** |
| --- | --- | --- | --- | --- | --- | --- |
| 301 | 2020 | **2.22** | Bacteroidetes | Flavobacteria | Flavobacteriales | **Flavobacteriaceae** |
| 750 | 3450 | **3.80** |  | Sphingobacteria | Sphingobacteriales | **Flexibacteraceae** |
| 216 | 2040 | **2.25** | Proteobacteria | Alphaproteobacteria | Rhizobiales | **Methylobacteriaceae** |
| 386 | 5589 | **6.15** |  |  |  | **Rhizobiaceae** |
| 475 | 9947 | **10.95** |  |  | Sphingomonadales | **unknown family** |
| 546 | 15948 | **17.56** |  |  |  | **Sphingomonadaceae** |
| 54 | 2076 | **2.29** |  | Gammaproteobacteria | Enterobacteriales | **Enterobacteriaceae** |
| 566 | 16383 | **18.04** |  |  | Pseudomonadales | **Pseudomonadaceae** |
| Remaining OTUs | 33362 | **36.74** |  |  |  | **others** |

The eight OTUs listed account for 63.26% of all sequences.
