# Supplementary material for: Distinct Phyllosphere Bacterial Communities on Arabidopsis Wax Mutant Leaves
Source: PLoS One. 2013 Nov 5;8(11):e78613. doi: 10.1371/journal.pone.0078613 (PMC3818481; doi:10.1371/journal.pone.0078613)
Supplement: Analysis S1 — Additional NMDS and ANOSIM Analyses of the amplicon dataset. Analysis of all OTUs which were present in more than one replicate of at least one plant line. (PDF) [file pone.0078613.s007.pdf]

We restricted a rarified amplicon dataset (rarefied to approx. 2300 sequences) to those OTUs which were present in more than one replicate of at least one plant line. This reduced the dataset to 175 OTUs, but still 98% of all sequences were recovered – all removed OTUs were present on only one replicate of one or more plant lines, and thus considered as atypical community members. Thus these OTUs were lost during rarification (44 OTUs) or due to their only sporadic appearance in the dataset (288 OTUs). We analysed this dataset based on relative abundance values of sequences. An NMDS analysis (stress: 0.2763) showed distinct clusters of the communities of the different plant lines. An ANOSIM analysis showed the bacterial communities of the five plant lines to be statistically significantly different. Due to the limited sample size, pairwise comparisons could only be made on a significance level of 0.1%. Under these circumstances the communities of Ler-*cer*6, Ler-*cer*9, Ler-*cer*16, *cer*1-*cer*9 and *cer*9-*cer*16 were found to be significantly different. The analyses were conducted using the Kulczynski similarity index.

NMDS plot:

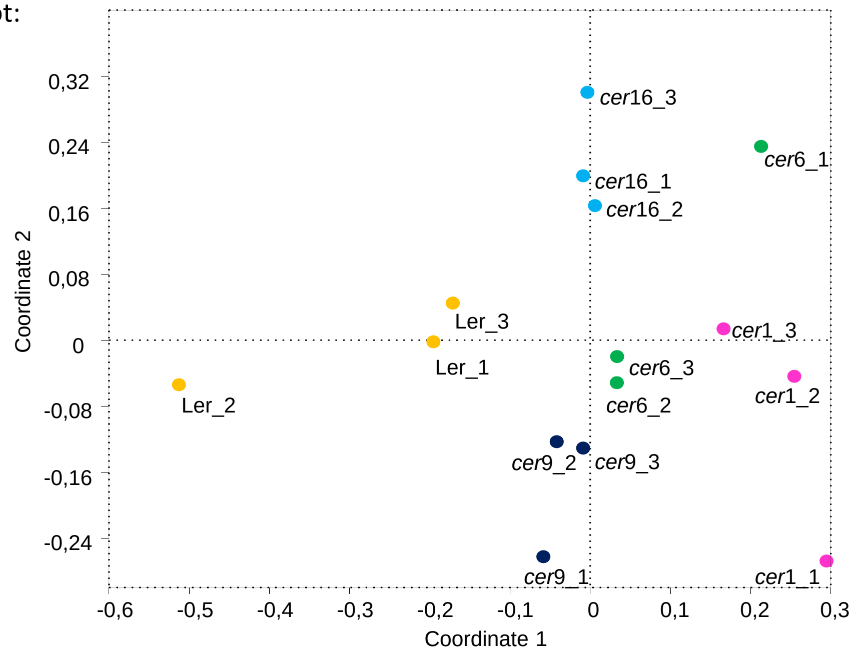

ANOSIM:

R = 0.7681		p = 0.0001		
R	Ler	*cer*1	*cer*6	*cer*9
Ler				
*cer*1	0.8519			
*cer*6	0.6667*	0.8519		
*cer*9	0.5926*	0.9259*	0.5926	
*cer*16	0.7593*	0.8519	0.8148	1*

In line 1 the overall test statistic is given for the comparison of all five groups. Underneath R-values for pairwise comparisons are shown. Asterisks at pairwise comparisons show the significance at the 0.1% level (lower significance levels could not be tested due to the small sample size (n=3 for each group)).
