# Supplementary material for: Distinct Phyllosphere Bacterial Communities on Arabidopsis Wax Mutant Leaves
Source: PLoS One. 2013 Nov 5;8(11):e78613. doi: 10.1371/journal.pone.0078613 (PMC3818481; doi:10.1371/journal.pone.0078613)
Supplement: Analysis S2 — Additional NMDS and ANOSIM Analyses of the amplicon dataset. Analysis of all OTUs which were present in more than one replicate of the dataset, regardless of the plant line. (PDF) [file pone.0078613.s008.pdf]

In order to broaden the perspective of our analysis, we analyzed the almost complete amplicon dataset statistically, irrespective of plant line-dependent community restrictions. We extracted all OTUs which were present in at least two of the 15 community replicates from the rarified dataset (rarefied to approx. 2300 sequences per replicate). This reduced the dataset to 251 OTUs and 97% of all sequences were recovered. OTUs were excluded only during rarification (44 OTUs) or were present on only one replicate in the whole dataset (212 OTUs). We analysed this dataset based on relative abundance values of sequences. An NMDS analysis (stress: 0.2779) showed distinct clusters of the communities of the different plant lines, apart from the *cer*6-replicates, which clustered in between the others. An ANOSIM analysis showed the bacterial communities of the five plant lines to be statistically significantly different. Due to the limited sample size, pairwise comparisons could only be made on a significance level of 0.1%. Under these circumstances the communities of Ler-*cer*1, Ler-*cer*9, Ler-*cer*16, *cer*1-*cer*6 and *cer*1-*cer*9 were found to be significantly different. The analyses were conducted using the Kulczynski similarity index.

NMDS plot:

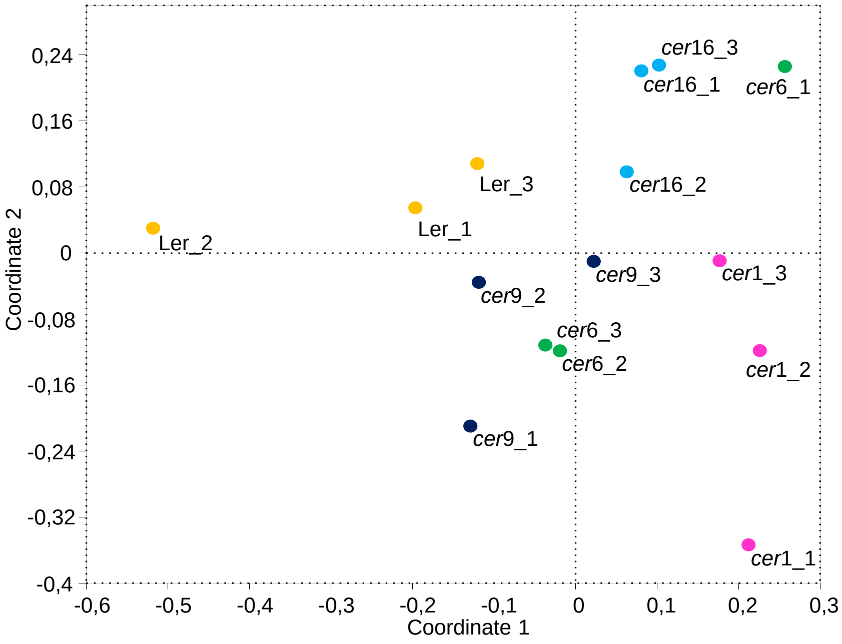

ANOSIM:

R = 0.6059		p = 0.0001		
R	Ler	*cer*1	*cer*6	*cer*9
Ler				
*cer*1	0.6667*			
*cer*6	0.5926	0.4444*		
*cer*9	0.5185*	0.7778*	0.2593	
*cer*16	0.7037*	0.7778	0.5926	0.963

In line 1 the overall test statistic is given for the comparison of all five groups. Underneath R-values for pairwise comparisons are shown. Asterisks at pairwise comparisons show the significance at the 0.1% level (lower significance levels could not be tested due to the small sample size (n=3 for each group)).
